# Predictability of 6 Intraocular Lens Power Calculation Formulas in People With Very High Myopia

**DOI:** 10.3389/fmed.2022.762761

**Published:** 2022-04-08

**Authors:** Yi-Ching Chu, Tzu-Lun Huang, Pei-Yao Chang, Wei-Ting Ho, Yung-Ray Hsu, Shu-Wen Chang, Jia-Kang Wang

**Affiliations:** ^1^Department of Ophthalmology, Far Eastern Memorial Hospital, New Taipei, Taiwan; ^2^Department of Electrical Engineering, Yuan Ze University, Taoyuan, Taiwan; ^3^Department of Medicine, National Taiwan University, Taipei, Taiwan; ^4^School of Medicine, National Yang Ming Chiao Tung University, Hsinchu, Taiwan; ^5^Department of Healthcare Administration and Department of Nursing, Oriental Institute of Technology, New Taipei, Taiwan

**Keywords:** optical biometry, intraocular lens, power calculation formulas, extremely high myopia, long axial length

## Abstract

**Purpose:**

To investigate the accuracy of 6 intraocular lens (IOL) power calculation formulas in predicting refractive outcomes in extremely long eyes.

**Setting:**

Department of Ophthalmology, Far Eastern Memorial Hospital, Taiwan.

**Design:**

Retrospective comparative study.

**Methods:**

In this retrospective single-center study, we reviewed 70 eyes of 70 patients with axial length (AL) ≥ 28 mm who had received an uneventful 2.2 mm corneal wound phacoemulsification and in-the-bag IOL placement. The actual postoperative refractive results were compared to the predicted refraction calculated with 6 formulas (Haigis, Hoffer Q, Holladay 1, SRK/T, T2, Barrett Universal II formulas) using IOLMaster 500 as optical biometry in the User Group for Laser Interference Biometry (ULIB) constants.

**Results:**

Overall, the Haigis and Barrett formulas achieved the lowest level of mean prediction error (PE) and median absolute error (MedAE). Hoffer Q, Holladay 1, SRK/T, and T2 had hyperopic prediction errors (*p* < 0.05). The Hoffer Q and Holladay 1 had significantly more MedAE between the 6 formulas. After the mean PE was zeroed out, the MedAE had no significant difference between each group. The absolute error tends to be larger in patients with longer AL. The absolute errors were 30.0–37.1% and 60.0–64.3% within 1.0 D of all patients compared to predicted refraction calculated using various formulas.

**Conclusion:**

The Haigis and Barrett Universal II formulas had a better success rate in predicting IOL power in high myopic eyes with AL longer than 28 mm using the ULIB constant in this study. The postoperative refractive results were inferior to the benchmark standards, which indicated that the precision of IOL power calculation in patients with high myopia still required improvement.

## Introduction

Myopia, which is commonly defined as a spherical equivalence (SE) of more than −0.5 D, is a worldwide health issue (1, 2). Over 22% of the global population has myopia, and approximately one-fifth to a quarter of myopic people have high myopia (SE>-5.0 D) (3, 4). In urban areas in Asia, such as China, Singapore, Korea, and Japan, 80–90% of students who complete high school are myopic, and 10–20% have high myopia (5, 6). In Taiwan, the prevalence of myopia is 60% in students under the ages of 12 years and over 85% in 12 to 18-year-olds. The prevalence of high myopia in 18-year-olds is approximately 16.9–20.8% (5).

A negative relationship exists between AL and myopia (7, 8). The average AL in emmetropic eyes is between 22–24 mm (9, 10). A 1 mm elongation of AL without other compensation is equivalent to a myopia shift of −2 or −2.5 D (11). Therefore, in patients with extremely high myopia of more than −10 D, the AL is usually more than 28 mm.

The accuracy of the intraocular lens (IOL) calculation depends primarily on the measurement of preoperative biometric data such as axial length (AL), corneal power (K), effective lens position (ELP) and the accuracy of IOL calculation formulas. A study based on preoperative and postoperative ultrasound biometry demonstrated that 54% of predictive refraction errors after IOL implantation can be attributed to AL, 38% to ELP, and 8% to K measurement error (12). ELP can be estimated by different IOL calculation formulas using variables such as preoperative AL, K, anterior chamber depth (ACD), lens thickness (LT), and white-to-white distance (WTW).

To improve the accuracy of IOL power calculation, different IOL calculation formulas were introduced. The third generation formulas, such as the Hoffer Q (13), Holladay 1 (14), and SRK/T (15), used two variables with AL and K to predict postoperative ACD. As for the T2 formula, it was modified to accommodate the calculation in patients with high myopia using only AL and K to estimate postoperative ACD (16). The Haigis formula, a fourth generation formula, used AL and measured ACD to calculate the ELP (17). The Barrett Universal II, also a fourth generation formula, incorporated five variables, namely AL, K, ACD, LT and WTW, to predict the ELP (18, 19).

The prediction of IOL power calculation was less accurate in high myopia patients with long eyes, which are commonly defined as AL longer than 24.5 mm (13). Previous studies report that the longer the AL, the more significant the deviation of IOL power (20, 21). The facts were relevant to some reasons such as the incorrect AL measurement due to poor eye fixation and the existence of posterior staphyloma, or the less predictability of IOL calculation formulas. The actual refractive error in extremely long eyes, often defined as AL > 28 mm, was sometimes over 1.00 diopter (D) compared to the refractive error predicted by formulas postoperatively, posing a great challenge for cataract surgeons (21, 22).

Our previous studies in 2008 (23) and 2013 (24) showed that the Haigis formula performed better than the Hoffer-Q, Holladay 1 and SRK/T formulas in longer eyes with AL ≥25.0 mm. The purpose of this study was to investigate the accuracy of 6 intraocular lens (IOL) calculation formulas in predicting refractive outcomes in eyes with AL equal to or more than 28.0 mm.

## Materials and Methods

The protocol of the study, which followed the principles of the Declaration of Helsinki, was approved by the institutional review board of the Far Eastern Memorial Hospital in Taiwan. All patients signed informed consent and agreed to receive cataract surgeries. The inclusion criteria were patients with cataract in the Far Eastern Memorial Hospital who underwent uneventful 2.2 mm clear corneal temporal incision phacoemulsification and IOL with in-the-bag placement by two surgeons (Wang JK and Chang SW) between January 2003 and December 2010. Axial length ≥ 28.0 mm and only one eye of each patient was included in the study. If the patient underwent cataract surgery of both eyes during this period, only the eye that was operated upon earlier was included.

Patients with the following conditions were excluded: patients with traumatic cataract, those with corneal pathology such as keratoconus, those who underwent prior keratoplasty or refractive surgery, those with intraoperative or postoperative complications, such as posterior capsular rupture or endophthalmitis, affecting the refractive result, and those not observed for at least 2 months after the surgery.

IOLMaster 500 (version 5.4, Carl Zeiss Inc., Berlin, Germany) as optical biometry was used to measure AL, K, and ACD preoperatively. ACD was measured from the corneal epithelium to lens. Auto-refraction and actual postoperative spherical equivalence (SE) were performed at a 1-month postoperative visit with a auto-refractor (Topcon AR, Topcon Inc., Tokyo, Japan) by experienced technicians.

The IOL calculation and SE predictions with the Haigis, Hoffer Q, Holladay 1 and SRK/T formulas were performed with the embedded software in IOLMaster 500 using the User Group for Laser Interference Biometry (ULIB) constant. The Barrett Universal II and T2 formulas were performed by the authors using the online table (Barrett https://calc.apacrs.org/barrett_universal2105/, accessed in May 2020 and December 2021, T2: http://www.richardsheard.net/T2Formula.aspx, accessed in May 2020 and December 2021) with variables measured by IOLMaster. For the T2 formula, ULIB SRK/T constants were used. For the Barrett formula, the A-constants were utilized for the selected IOL that we could access on the website; and if the selected lens were not available on the website, then ULIB SRK/T constants were used for IOL power calculations.

The power of the implanted IOL in our routine clinical service was chosen according to the Haigis formula. The actual postoperative SE was compared with the predicted postoperative SE using each formula. The mean prediction error (PE) was defined as the average of the differences between the actual and the predicted SE of the postoperative refractive error.

We zeroed out the PE in the following manner: first, we calculated the mean PE of each formula; next, we calculated a new predictive postoperative SE by adding the mean PE from the original predictive postoperative SE of each subject. Then, we calculated a zeroed out PE by subtracting the new predictive postoperative SE from the actual postoperative SE. After adjusting the mean PE to zero, the mean absolute error (MAE), defined as the mean absolute value of prediction error, was calculated.

Since absolute errors are not a Gaussian distribution, median absolute errors (MedAE), the median absolute value of prediction error, were calculated. The differences in the MedAE according to the IOL calculation formulas were analyzed. The percentage of the patients with PE within ± 0.50 D and ± 1.00 D was then evaluated. All patients were divided into three groups based on their eye's AL: 28.00~28.99 mm as Group A, 29.00~30.99 mm as Group B, and AL ≥ 31 mm as Group C.

SPSS ver. 22.0 (SPSS Inc., Chicago, IL, USA) was used for statistical analysis. The differences in absolute error between each formula were assessed using the Friedman test. If a significant difference was noticed in the Friedman test, Bonferroni correction was performed further to compare the MedAE of each formula with that of the Haigis. The percentage of patients' eyes within ± 0.50D and ± 1.00D of PE were compared using Cochran's *Q* test. The correlation between AL and absolute error was evaluated using the univariate simple linear regression model. A statistically significant difference was defined as a *p*-value < 0.05.

## Results

A total of 70 eyes of 70 patients were included in the study. The mean age of the patients was 55.21 years. The implanted IOL power ranged from −8.0 to +20.0 D and the mean power was 29.86 ± 1.59 D. The mean AL was 29.86 ± 1.59 mm, and the mean horizontal K was 42.29 ± 1.96 D and the mean vertical K was 43.66 ± 2.24 D. There were 24 patients in Group A, 31 in Group B, and 15 in Group C. Various clinical data relating to the patients and the 3 subgroups are listed in Table 1. Only AL and implanted IOL power were significantly different between the 3 subgroups. Age, K, and ACD were matched between the subgroups. The IOLs used in the study were the 3-piece AcrySof MA60MA, the 1-piece AcrySof SA60AT, SN60AT, and SN60WF (Alcon Inc.), the 1-piece Superflex 920H and M-flex 630F (Rayner Intraocular Lens Limited), and the 1-piece Tecnis ZCB00 (Abbott Medical Optics, Johnson & Johnson Vision). The types of IOLs that were implanted are listed in Table 2.

**Table 1:** The clinical data of all patients and 3 subgroups.

	**All groups**	**Group A**	**Group B**	**Group C**	
**Parameter**	***n =* 70**	***n =* 24**	***n =* 31**	***n =* 15**	***p***
Age (years)	55.21 ± 12.02	55.83 ± 13.30	54.39 ± 12.51	55.93 ± 8.07	0.88
AL (mm)	29.86 ± 1.59	28.48 ± 0.28	29.68 ± 0.47	32.43 ± 1.24	<0.001*
Implanted IOL power (D)	6.24 ± 5.57	9.94 ± 2.97	6.45 ± 4.42	−0.13 ± 5.32	<0.001*
K1 (D)	42.29 ± 1.96	41.82 ± 1.24	42.50 ± 2.46	42.61 ± 1.54	0.35
K2 (D)	43.66 ± 2.24	42.80 ± 1.62	43.94 ± 2.64	44.42 ± 1.76	0.06
ACD (mm)	3.50 ± 0.44	3.49 ± 0.36	3.59 ± 0.52	3.33 ± 0.30	0.16

AL, axial length; K1, horizontal corneal power; K2, vertical corneal power; ACD, anterior chamber depth (measured from corneal epithelium to lens); IOL, intraocular lens; D, diopter; mm, millimetre. Group A: 28.00 mm ≤ AL < 29.00 mm; Group B: 29.00 mm ≤ AL < 31.00 mm; Group C: AL ≥ 31.00 mm. *P < 0.05, Significant difference between each groups.

**Table 2:** Intraocular lens implantations by IOL model (*n* = 70).

**IOL Model**	**Implantations (n)**	**Eyes (%)**
Alcon Acrysof SA60AT	13	18.57
Alcon Acrysof MA60MA*	21	30
Alcon Acrysof SN60WF	17	24.29
Rayner Superflex 920H†	5	7.14
Rayner M-flex 630F	2	2.86
AMO Tecnis ZCB00	11	15.71
Alcon Acrysof SN60AT	1	1.43

IOL, intraocular lens. *Including 10 IOLs with negative power and 1 with zero power. †Including 1 IOL with negative power.

**Figure 1:**
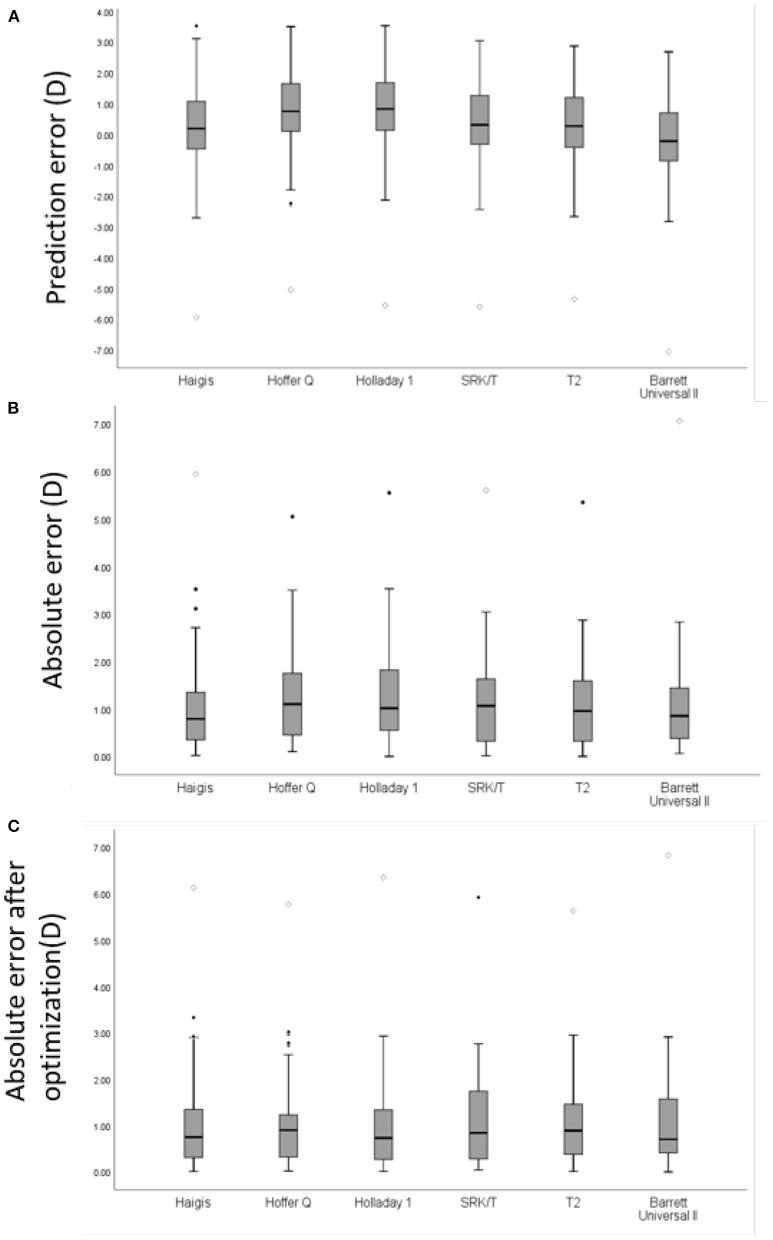
A box plot of mean refractive error of intraocular lens power prediction in all eyes with AL≥28.0 mm. **(A)** Prediction error (PE) of each formula. **(B)** Absolute error (AE) of each formula in all eyes. **(C)** AE of each formula in all eyes after zeroing out the mean PE. Round mark (•) means outlier values between 1.5 to 3 box lengths from either end of the box; diamond mark (♢) means extreme values that are more than 3 box lengths from either end of the box.

In all patients, the mean PE of the Haigis was 0.19 D, and the Barrett formula was −0.23 D, which was close to zero (*p* > 0.05). The Hoffer Q, Holladay 1, SRK/T, and T2 formulas had a significantly hyperopic mean PE (*p* < 0.05). The Haigis formula obtained the lowest MedAE, which was 0.79D, followed by the Barrett formula with 0.86D, the T2 formula with 0.96 D, the Hoffer Q formula with 1.00 D, the Holladay 1 formula with 1.02 D and the SRK/T formula with 1.07 D. The MedAE generated by the Haigis formula was comparable to those by the T2, and Barrett formulas but was significantly lower than those by the Hoffer Q and Holladay 1 formulas (*p* < 0.05). After adjusting the PE to zero, the MedAE had no significant difference between each formula. The distribution of refraction error is presented in Figure 1. The prediction errors within ± 0.50 D were 30.0–37.1% and within ± 1.00 D were 60.0–64.3% using various formulas, without significant difference between each formula (*p* = 0.124 within 0.50 D and *p* = 0.895 within 1.00 D) (Table 3).

**Table 3:** Postoperative refractive results of all patients.

	**Before adjusting Mean PE**					**After adjusting Mean PE to zero**			
**Formula**	**Mean PE ±SD (D)**	**Range (D)**	**MAE ±SD, MedAE (D)**	**p1**	**p1'**	**MAE ±SD, MedAE (D)**	**p2**	**The proportions of the eyes with PE within** **±0.50 D and** **±1.00 D (%)**	
								**±0.50D**	**±1.00D**
Haigis	0.19 ± 1.49	−5.94, 3.52	1.06 ± 1.02, 0.79	<0.001		1.05 ± 1.02, 0.75	0.49	34.3	62.9
Hoffer Q	0.72 ± 1.46*	−5.05, 3.50	1.29 ± 1.03, 1.00†		0.003†	1.07 ± 1.00, 0.90		31.4	64.3
Holladay 1	0.80 ± 1.45*	−5.55, 3.53	1.29 ± 1.03, 1.02†		0.003†	1.02 ± 1.03, 0.73		37.1	64.3
SRK/T	0.43 ± 1.51*	−5.60, 3.04	1.13 ± 1.00, 1.07		1.000	1.10 ± 1.00, 0.84		31.4	61.4
T2	0.28 ± 1.48*	−5.35, 2.87	1.12 ± 0.98, 0.96		1.000	1.10 ± 0.97, 0.89		30.0	62.9
Barrett	−0.23 ± 1.53	−7.06, 2.68	1.10 ± 1.06, 0.86		1.000	1.08 ± 1.06, 0.71		32.9	60.0

ULIB, The User Group for Laser Interference Biometry; MAE, mean absolute error; MedAE, median absolute error; PE, predictive error; D, dioptre. *Significantly different from the mean PE of zero (p < 0.05). p1, p-value of the MedAE by Friedman test among all formulas. p1', adjusted p-value by Bonferroni correction with the MedAE of each formula with that of the Haigis. p2, p-value of the MedAE by Friedman test among all formulas after adjusting mean PE to zero. †Significantly different from the MedAE of Haigis (p < 0.05).

In Group A, the mean PE of the Haigis, SRK/T, T2, and Barrett formulas were 0.06 D, 0.21D, 0.04 D, and −0.08 D, respectively, which were close to zero (*p*>0.05). The Hoffer Q and Holladay 1 formulas had significantly hyperopic mean PE (*p* < 0.05). The T2 formula obtained the lowest MedAE, which was 0.81 D, followed by the Haigis and SRK/T formulas with a MedAE of 0.86 D, the Barrett with 0.88 D, the Holladay 1 with 1.00 D, and the Hoffer Q with 1.20 D. There was no significant difference in the MedAE between each group before and after adjusting the PE to zero (*p* = 0.485 and 0.49, respectively). The prediction errors after adjusting the mean PE to zero within ± 0.50 D were 30.0–37.1%, and within ± 1.00 D were 60.0–64.3% of Group A using various formulas, without significant difference between each formula (*p* = 0.923 in ± 0.50 D and *p* = 0.514 in ± 1.0 D) (Table 4).

**Table 4:** Postoperative refractive results of Group A (29.00 mm>AL≥28.00 mm).

	**Before adjusting mean PE**				**After adjusting mean PE to zero**			
**Formula**	**Mean PE ±SD (D)**	**Range (D)**	**MAE ±SD, MedAE (D)**	**p1**	**MAE ±SD, MedAE (D)**	**p2**	**The proportions of the eyes with PE within** **±0.50 D and** **±1.00 D (%)**	
							**±0.50D**	**±1.00D**
Haigis	0.06 ± 1.31	−2.69, 2.45	1.05 ± 0.79, 0.86	0.485	1.05 ± 1.02, 0.75	0.49	34.3	62.9
Hoffer Q	0.42 ± 1.34*	−2.30, 2.70	1.16 ± 0.80, 1.20		1.07 ± 1.00, 0.90		31.4	64.3
Holladay 1	0.66 ± 1.29*	−1.93, 2.91	1.20 ± 0.81, 1.00		1.02 ± 1.03, 0.73		37.1	64.3
SRK/T	0.21 ± 1.30	−2.28, 2.73	1.01 ± 0.79, 0.86		1.10 ± 1.00, 0.84		31.4	61.4
T2	0.04 ±1.33	−2.67, 2.58	1.05 ± 0.85, 0.81		1.10 ± 0.97, 0.89		30.0	62.9
Barrett	−0.08 ± 1.33	−2.72, 2.15	1.09 ± 0.76, 0.88		1.08 ± 1.06, 0.71		32.9	60.0

ULIB, The User Group for Laser Interference Biometry; MAE, mean absolute error; MedAE, median absolute error; PE, predictive error; D, dioptre. *Significantly different from the mean PE of zero (p < 0.05). p1, p-value of the MedAE by Friedman test among all formulas. p2, MedAE by Friedman test among all formulas after adjusting mean PE to zero.

In Group B, the mean PE of the Haigis, SRK/T, T2, and Barrett formulas were 0.37 D, 0.47 D, 0.40 D, and −0.07 D, respectively, which were close to zero (*p* > 0.05). The Hoffer Q and Holladay 1 formulas had significantly hyperopic mean PE (*p* < 0.05). The Haigis formula obtained the lowest MedAE, which was 0.49 D, followed by the SRK/T with a MedAE of 0.64 D, the T2 with 0.65 D, the Holladay 1 with 0.81 D, the Hoffer Q and the Barrett with 0.85 D. The MedAE generated by the Haigis formula was comparable to those by the SRK/T, and T2 formulas but was significantly lower than those by the Hoffer Q and Holladay 1 formulas (*p* < 0.05). There was no significant difference in the MedAE between each group before and after adjusting the PE to zero (*p* = 0.078 and 0.082, respectively). The prediction errors within ± 0.50 D were from 25.8-51.6% and within ± 1.00 D were from 58.1%-64.5% of Group B using various formulas without significant difference between each formula (*p* = 0.832 within 0.50 D and *p* = 0.429 within 1.00 D) (Table 5).

**Table 5:** Postoperative refractive results of Group B (31.00≥AL>29.00).

	**Before adjusting mean PE**					**After adjusting Mean PE to zero**			
**Formula**	**Mean PE ±SD (D)**	**Range (D)**	**MAE ±SD, MedAE (D)**	**p1**	**p1'**	**MAE ±SD, MedAE (D)**	**p2**	**The proportions of the eyes with PE within** **±0.50 D and** **±1.00 D (%)**	
								**±0.50D**	**±1.00D**
Haigis	0.37 ± 1.31	−2.71, 3.52	0.96 ± 0.97, 0.49	0.042		0.96 ± 0.91, 0.66	0.984	51.6	64.5
Hoffer Q	0.88 ± 1.26*	−2.24, 3.50	1.17± 0.99, 0.85†		0.012†	0.92 ± 0.87, 0.62		35.5	61.3
Holladay 1	1.00 ± 1.28*	−2.13, 3.53	1.25 ± 1.04, 0.81†		0.012†	0.96 ± 0.88, 0.72		25.8	58.1
SRK/T	0.47 ± 1.30	−2.44, 3.04	1.02 ± 0.94, 0.64		0.436	0.99 ± 0.86, 0.76		45.2	58.1
T2	0.40 ±1.26	−2.65, 2.86	0.98 ± 0.89, 0.65		0.821	0.96 ± 0.82, 0.73		45.2	61.3
Barrett	−0.07 ± 1.26	−2.83, 2.68	0.97 ±0.81, 0.85		0.688	0.95 ±0.85, 0.63		32.3	64.5

*ULIB: The User Group for Laser Interference Biometry; MAE: mean absolute error; MedA: median absolute error; PE: predictive error; D: diopter*.

*Significantly different from the mean PE of zero (p < 0.05).

*p1: p-value of the MedAE by Friedman test among all formulas*.

*p1': adjusted p-value by Bonferroni correction with the MedAE of each formula with that of the Haigis*.

*p2: p-value of the MedAE by Friedman test among all formulas after adjusting mean PE to zero †Significantly different from the MedAE of Haigis (p < 0.05)*.

In Group C, the mean PE of the Haigis, T2, SRK/T and Barrett formulas were 0.01, 0.41, 0.21, and −0.79, respectively, which were close to zero (*p* > 0.05). The Hoffer Q and Holladay 1 formulas had significantly hyperopic mean PE (*p* < 0.05). The Barrett formula obtained the lowest MedAE, which was 0.75 D, followed by the Haigis with a MedAE of 0.89 D, the SRK/T with 1.13 D, the T2 with 1.20 D, the Holladay 1 with 1.4 D, and the Hoffer Q with 1.65 D. Using the ULIB constant, the MedAE generated by the Haigis was comparable to those by the SRK/T, T2 and Barrett formulas but was significantly lower than those by the Hoffer Q and Holladay 1 formulas (*p* < 0.05). After the mean PE was zeroed out, the MedAE had no significant difference between each formula. The prediction errors within ± 0.50 D were 12.5–37.5% and within ± 1.00 D were 31.3–62.5% of Group C using various formulas (Table 6).

**Table 6:** Postoperative refractive results of group C (AL>31.00mm).

	**Before adjusting mean PE**				**After adjusting mean PE to zero**			
**Formula**	**Mean PE ±SD (D)**	**Range (D)**	**MAE ±SD, MedAE (D)**	**p1**	**MAE ±SD, MedAE (D)**	**p2**	**The proportions of the eyes with PE within** **±0.50 D and** **±1.00 D (%)**	
							**±0.50D**	**±1.00D**
Haigis	0.01 ± 1.88	−5.94, 2.28	1.31 ± 1.35, 0.89	0.078	1.25 ± 1.42, 0.70	0.082	20.0	60.0
Hoffer Q	0.87 ± 1.90*	−5.05, 3.25	1.74 ± 1.16, 1.65		1.37 ± 1.33, 0.98		13.3	26.7
Holladay 1	0.59 ± 1.89*	−5.55, 2.79	1.53 ± 1.26, 1.40		1.14 ± 1.53, 0.68		13.3	33.3
SRK/T	0.21 ± 2.01	−5.60, 2.73	1.54 ± 0.30, 1.13		1.41 ± 1.44, 0.82		13.3	26.7
T2	0.41 ± 1.93	−5.35, 2.87	1.53 ± 1.25, 1.20		1.44 ± 1.30, 0.92		20.0	20.0
Barrett	−0.79 ± 2.04	−7.06, 1.44	1.39 ± 1.69, 0.75		1.36 ± 1.62, 0.90		40.0	64.5

ULIB, The User Group for Laser Interference Biometry; MAE, mean absolute error; MedAE, median absolute error; PE, predictive error; D, dioptre. *Significantly different from the mean PE of zero (p < 0.05). p1, p-value of the MedAE by Friedman test among all formulas. p2, MedAE by Friedman test among all formulas after adjusting mean PE to zero.

**Figure 2:**
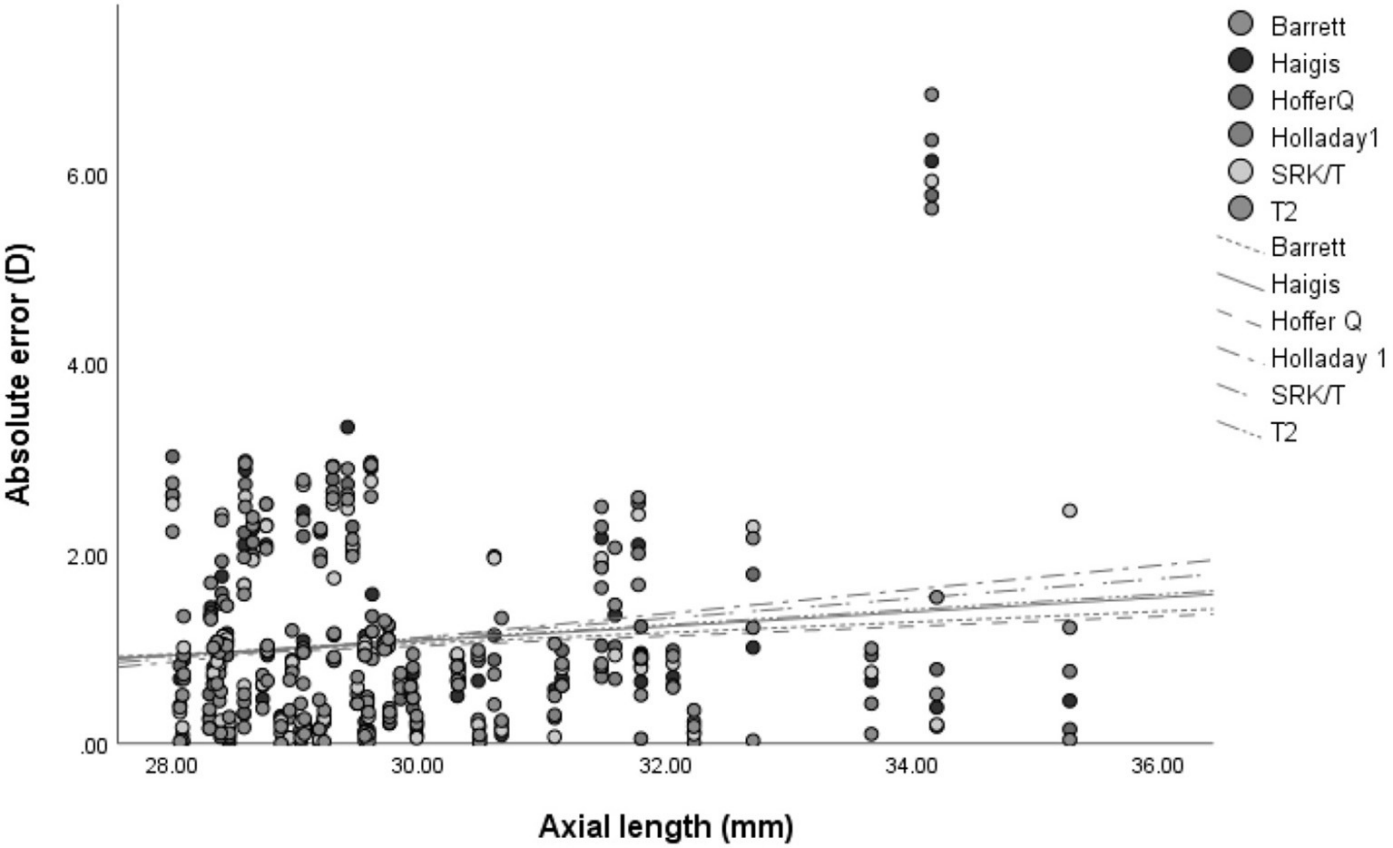
The association between the absolute error of refractive outcome and axial length in 6 intraocular lens calculation formulas.

As AL increased, so did the predictive error (MedAE). This is shown in Figure 2. However, no significant correlation between AL and absolute error was noted after zeroing out the PE in all formulas (*p* > 0.05) (Table 7).

**Table 7:** The association between absolute error and axial length using simple regression model.

	**r**	**P**
Haigis	0.087	0.473
Hoffer Q	0.121	0.317
Holladay 1	0.080	0.509
SRK/T	0.201	0.094
T2	0.172	0.156
Barrett	0.120	0.323

## Discussion

The postoperative refractive result, highly affected by the accurate calculation of intraocular lens (IOL) power, played an essential role in patients' satisfaction with their cataract surgery (25).

The average age of our patients was 55.23 years. In recent studies on highly myopic eyes with mean AL of 29 mm (for instance, Rong's study with mean AL of 29.3 mm, and Zhou's study with mean AL of 29.63 mm), the average age was 62.15 and 65.23, respectively. This was much younger than the usual average age of patients studied (26). Myopic eyes develop cataracts at an earlier age as first evidenced and proven in 1980 by Hoffer (27). Additionally, myopia was associated with an increased prevalence of nuclear and posterior subcapsular cataracts (28). The age of the patients in our study with AL of 29.92 was, therefore, significantly less than that reported in previous studies.

In a previous study, Liu et al. found no significant correlations between PE and AL for other formulas (29). Many studies have focused on high myopic eyes with AL> 25 or 26 mm and compared them with extremely high myopic eyes (20, 30–32). In their study, El-Nafees et al. classified the AL into 25–27 mm, 27–29 mm, and ≥29 mm and found that the SRK/T, Barrett and Holladay formulas performed equally (32). Rong et al. classified the AL into 28–30 mm and ≥30 mm and found that the Barrett formula performed better in the AL>30 mm category (30). In their study, Zhou et al. categorized the AL as 27–30 mm (*n* = 47) and AL ≥30 mm and found that the Barrett formula performed better in all groups (20). Chen et al. classified AL into 28–30 mm, 30.01–33 mm, and >33.01 mm and found that the SRK/T and Haigis performed better in Chinese patients whose eyes had an AL ranging between 26 and 33 mm (21). Lin found that the accuracy of the SRK/T was better than Haigis in AL of 28–30 mm but less accurate in AL >30 mm (31). Zhang found that the SRK/T and Haigis were both accurate in AL >29 mm (33). With even longer ALs (approximately 31.0 to 32.0 mm), the required calculation of IOLs may change from plus to minus power, causing more difficulties in the selection of IOL power (22, 34). Therefore, we selected the cutoff values as 28–29 mm, 29–31 mm, and >31 mm.

In our study, we found that the Haigis and Barrett Universal II formulas had the average predictive refractive error nearest to zero and the lowest mean and median absolute refractive error between all formulas. This was for eyes with AL more than 28 mm using ULIB constants. Our results showed that the Haigis and Barrett formulas performed better, and these results were consistent with previous studies on long eyes. Bang et al. showed that the Haigis and SRK/T formulas had a more accurate prediction of IOL power in eyes with AL ≥27 mm (35). The study by Melles et al. revealed that the Barrett formula performed better than the Haigis, SRK/T, Hoffer Q and Holladay 1 formulas for eyes with AL ≥ 28 mm (36).

In our subgroup analysis, the Haigis, SRK/T, T2, and Barrett formulas had better predictive accuracy for eyes with AL longer than 31 mm using ULIB constants. Bang et al. showed that the Haigis formula performed better than the SRK/T formula in AL ≥ 29.06 mm (35). Zhang et al. demonstrated that the SRK/T and Haigis formulas had similar predictive outcomes in eyes with AL ≥ 29 mm using ULIB constants (37). Chen et al. found that the Haigis and SRK/T formulas had less absolute error compared to the Holladay 1 and Hoffer Q formulas in eyes with AL of 28–33 mm, but the Haigis formula performed the best in eyes with AL ≥ 33 mm (21). Rong et al. found that, in eyes with AL ≥28 mm, the Barrett and Haigis formulas had similar performance, but the Barrett formula performed better in eyes with AL ≥ 30 mm (30). Zhou et al. found that the Barrett formula had the lowest MAE compared to the Haigis, SRK/T, Holladay, and Hoffer Q formulas for eyes with AL of 27–30 mm, but the Barrett and Haigis formulas performed equally better than others in AL ≥ 30 mm (20). Our study results agree with these studies on extremely long eyes that the Barrett, T2, and Haigis formulas have a superior performance among the 6 formulas.

The Hoffer Q and Holladay 1 formulas produced a significantly postoperative hyperopic shift and higher average and median absolute errors in all categories of long eyes in this research. Aristodemou et al. tested the accuracy of the Hoffer Q, Holladay 1 and SRK/T formulas in 8,108 eyes, and they found that the Hoffer Q formula was the most accurate formula for patients with AL <21 mm, and the Holladay 1 formula had the highest accuracy in eyes between 23.5 and 26.0 mm (38). However, in long eyes, these 2 formulas did not predict the IOL power accurately. In a review article by Hoffer, the Barrett, Haigis, Olsen, and SRK/T formulas were better than the Hoffer Q and Holladay 1 formulas in the group of AL > 26 mm (39). Our results were consistent with the above findings that the Hoffer Q and Holladay 1 formulas had poor predictive performance in patients with high myopia.

The T2 formula was developed by Sheard et al. to correct the non-physiologic behavior of the SRK/T formula (16). According to their report, the T2 formula can be directly substituted for the SRK/T formula, improving the refractive outcomes by 10%.

In our study, however, we discovered that for IOL power in long eyes, the SRK/T and T2 formulas possessed similar predictive abilities. Previous studies have shown contradictory results in the performance of both formulas regarding patients with high myopia. Kane et al. demonstrated that the SRK/T formula performed better than the T2 formula in eyes with AL >26 mm (40). Similarly, Idrobo et al. concluded that the SRK/T formula was superior to the T2 formula in eyes with AL longer than 25 mm (41). However, Cooke et al. found that the T2 formula had better accuracy than the SRK/T formula in the group of AL ≥ 25.97 mm (42). The contradictory results could be attributed to their small sample size of highly myopic eyes.

To eliminate systemic error caused by the lens factor, we zeroed out the mean PE to zero. After the mean PE were zeroed out, the MedAE displayed no significant difference between each formula. It could be because of factors such as fewer number of eyes with extremely long axial length, various types of IOL, and surgeon bias. In extremely high myopia, the lens factor selection may need to be modified from ULIB constants and requires further investigation.

This implies that AL elongation can lead to the inaccuracy in IOL formula prediction. Roessler et al. found that, in eyes with AL of more than 26.5 mm, the absolute predictive error using the Haigis formula increased significantly with longer AL values (r = 0.61, *p* < 0.001) (43). The study by Zhou et al. showed a positive correlation between AL and predictive error in eyes with AL > 24.5 mm (20). Similarly, research by Chen et al. demonstrated that higher absolute error was associated with longer AL in eyes with AL above 28 mm while using the SRK/T and Hoffer Q formulas (*r* = 0.212 and 0.213 respectively, *p* < 0.05) (21). A similar trend of higher absolute errors in long eyes of patients with high myopia was noted in our analysis, but no significant correlation between AL and mean or median absolute errors in any formula was observed. This could be because of the smaller sample size in different AL groups that might have interfered with the performance of the simple linear regression.

Prediction errors of ± 0.50 D and ± 1.00 D are clinically important as it might be associated with postoperative satisfaction in patients. Several studies defined benchmark standards for refractive outcomes in cataract surgery. Gale et al. used IOLMaster 500 for biometry measurement and used the optimizing constant for IOL calculation and set up a benchmark standard with 85% of patients achieving within 1.0 D of the predicted value, and with 55% of patients within 0.5 D for the National Health Service of the United Kingdom in 2009. This was revised to a higher level of 88.76% within 1 D and 62.36% within 0.5 D in 2019 (44, 45). In 2011, Hahn et al. developed a benchmark standard of 80% of patients, achieving the final predictive error within 0.5 D through a multicenter study in Germany using standardized A constant with IOLMaster 500 (45). Similarly, in 2014, Simon et al. submitted a benchmark standard of 94% of patients in the United States, achieving within 1.0 D of target refraction after cataract surgery using IOLMaster 500 (46). The benchmark standards listed above were based on eyes with different AL. However, in our study on eyes with very long AL (more than 28 mm), the prediction errors were 30.0–37.1% within ± 0.50 D and 60.0–64.3% within ± 1.00D, which were all below the proposed benchmark standards. Furthermore, since the IOL powers are offered in 1.0 D increments in the extreme range of power, this could result in lower satisfaction in the postoperative refraction. The inferior refractive outcomes in patients with high myopia require a detailed analysis before the cataract surgery, and additional effort is needed to develop more precise IOL calculation formulas for these patients.

## Limitations

There were some limitations in our study. First, since the eyes with AL greater than 28 mm were uncommon, only a small number of recruited subjects was included in our study. Second, in addition, we used AL, K, and ACD to calculate the Barrett Universal II formula, and the lack of LT and WTW might have influenced the accuracy in IOL power calculation. However, a recent study showed high agreement in IOL power difference while omitting WTW and LT in the Barrett Universal II formula (47). Third, we enrolled patients operated on by two surgeons, and this probably introduced surgeon bias factors. Fourth, we did not choose a certain IOL for evaluation, which may have caused bias due to different IOL constants. Although the results were possibly stronger in the single IOL type studies, the variety of IOLs used in this study can represent a real-world situation in clinical practice. Moreover, this study was designed as a retrospective comparative and a single-center study with limited external validity.

## Conclusions

In conclusion, the Haigis and Barrett Universal II formulas had a better success rate in predicting IOL power in high myopic eyes with AL longer than 28 mm using the ULIB constant in this study. The predictive refractive error could increase in longer eyes. The postoperative refractive results did not meet the benchmark standards in all formulas in this study. The results indicate that the precision of IOL power calculation is less accurate in extremely long eyes, and the refractive surprise should be borne in mind prior to surgery.

## Value Statement

### What Was Known

- Several modern formulas have demonstrated a superior ability to calculate intraocular lens (IOL) power despite limited evidence from past publications relating to patients with extremely high myopia.
- Previous studies have received controversial results when comparing the performance of the SRK/T and T2 formulas in myopic eyes, and the outcomes of both formulas in extremely highly myopic eyes were unsatisfactory.

### What This Paper Adds

- The Haigis and Barrett Universal II formulas can offer reliable IOL calculation formulas for long eyes with axial length (AL) ≥ 28 mm, even with AL ≥ 31 mm.
- The T2 and SRK/T formulas offer similar predictive ability for IOL power in patients with AL ≥ 28 mm.

## Author's Note

Research relating to the predictability of intraocular lens power calculation formulas in patients with very high myopia is uncommon. Additionally, in this study, we have investigated the accuracy of some new formulas.

## Data Availability Statement

The original contributions presented in the study are included in the article/supplementary material, further inquiries can be directed to the corresponding author/s.

## Ethics Statement

The studies involving human participants were reviewed and approved by Institutional Review Board of Far Eastern Memorial Hospital. The patients/participants provided their written informed consent to participate in this study.

## Author Contributions

Y-CC and J-KW designed the study, collected the clinical data, performed the statistics, wrote the main manuscript text, and prepared tables. S-WC, T-LH, P-YC, W-TH, Y-RH, and J-KW reviewed, corrected, and approved the manuscript. All authors contributed to the article and approved the submitted version.

## Funding

This study was supported by grants from the Far Eastern Memorial Hospital (FEMH-YZU-2021-008), Taiwan.

## Conflict of Interest

The authors declare that the research was conducted in the absence of any commercial or financial relationships that could be construed as a potential conflict of interest.

## Publisher's Note

All claims expressed in this article are solely those of the authors and do not necessarily represent those of their affiliated organizations, or those of the publisher, the editors and the reviewers. Any product that may be evaluated in this article, or claim that may be made by its manufacturer, is not guaranteed or endorsed by the publisher.
